# The clinical application of nigrosome 1 detection on high-resolution susceptibility-weighted imaging in the evaluation of suspected Parkinsonism: The real-world performance and pitfalls

**DOI:** 10.1371/journal.pone.0231010

**Published:** 2020-04-02

**Authors:** Jungbin Lee, A. Leum Lee, Jeong-Ho Park, Ji Eun Moon, Jung-Mi Park, Sang joon Kim, Kee-Hyun Chang

**Affiliations:** 1 Department of Radiology, Soonchunhyang University Bucheon Hospital, Bucheon, Korea; 2 Department of Neurology, Soonchunhyang University Bucheon Hospital, Bucheon, Korea; 3 Department of Biostatistics, Clinical Trial center, Soonchunhyang University Bucheon Hospital, Bucheon, Korea; 4 Department of Nuclear medicine, Soonchunhyang University Bucheon Hospital, Bucheon, Korea; 5 Department of Radiology, Asan Medical Center, Seoul, Korea; 6 Department of Radiology, Human Medical Imaging and Intervention Center, Seoul, Korea; University at Buffalo, UNITED STATES

## Abstract

**Purpose:**

To evaluate the real-world diagnostic performance of high-resolution susceptibility-weighted imaging (HR-SWI) and investigate whether the reader’s predictions can be used to find cases where HR-SWI finding and final clinical diagnosis matched.

**Methods:**

This retrospective study enrolled patients with suspected Parkinsonism (n = 48) or volunteers with other intracranial pathologies (n = 31) who underwent brain magnetic resonance imaging (MRI) including HR-SWI, which was used to evaluate nigrosome 1 (NG1). All patients with suspected Parkinsonism underwent N-3-fluoropropyl-2-carbomethoxy-3-4-iodophenyl nortropane (FP-CIT) positron emission tomography and a clinical diagnosis was made by a neurologist. The HR-SWI data were qualitatively analyzed by two independent reviewers. A consensus reading was performed and a diagnostic confidence score was assigned. According to final clinical diagnosis, diagnostic sensitivity, specificity, and accuracy were calculated. Receiver operating characteristic (ROC) curve analysis was used to examine whether the diagnostic confidence score could be used to identify HR-SWI finding—final clinical diagnosis matched cases.

**Results:**

Of the 48 patients with suspected Parkinsonism, 31 were diagnosed with idiopathic Parkinson’s disease, and three with multiple system atrophy. The remaining 14 patients were included in the disease control group. Of the 31 volunteers, 10 subjects were excluded due to possibility of nigrostriatal degeneration and finally 21 subjects were enrolled as controls with non-Parkinsonism pathology (non-PD control). After consensus reading, 25 subjects were classified as true positive and 28 as true negative, according to HR-SWI findings. The calculated diagnostic sensitivity, specificity, and accuracy were 73.5%, 80.0%, and 76.8%, respectively. With using diagnostic concordance score, the area under the ROC curve for the detection of concordance case was 0.83 (95% CI: 0.72–0.91, p < 0.05).

**Conclusion:**

The diagnostic performance of NG1 detection using HR-SWI with 3T MRI was within acceptable range. Using the reader's diagnostic confidence could be helpful to find cases which HR-SWI finding and final clinical conclusion match. So HR-SWI may be of added value in the evaluation of suspected Parkinsonism.

## Introduction

Nigrosome 1 (NG1) is the largest cluster of dopaminergic cells, which show maximum depletion in Parkinson’s disease [1, 2]. It is located in dorsolateral aspect of substantia nigra pars compacta, and initially there have been attempts to observe it with 7T magnetic resonance imaging (MRI) with academic purpose [3, 4]. In subsequent studies, detection of NG1 using clinical 3T MRI showed high accuracy in predicting Parkinson’s disease [5–8].

In recent years, there have been many studies evaluating the accuracy of Parkinson’s disease diagnosis using HR-SWI in various clinical settings. NG1 detection using HR-SWI is known to be useful for screening patients with drug-induced Parkinsonism [9]. Parkinson-plus syndromes are also known to show abnormalities on HR-SWI [6, 10]. In addition, the clinical laterality in early idiopathic Parkinson’s disease and the laterality of NG1 abnormalities on HR-SWI are highly correlated [11].

However, technically, imaging parameters and protocols for NG1 detection are not standardized [1]. By the definition of the term “high resolution”, recent studies suggest a minimum spatial resolution of 0.5 x 0.5 x 1.0mm or 0.67 x 0.67 x 1.34 mm to evaluate NG1 on susceptibility weighted images including SWI or QSM [1, 12]. In terms of image contrast, according to Kim et al., the use of susceptibility map-weighted imaging (SMWI) allows for quantitative assessment and enhances the diagnostic performance by increasing the contrast-to-noise ratio (CNR) [1, 13]. Unfortunately, this technique requires a post processing step with commercial software, so further resource consumption must be considered to apply it in routine practice. On the contrary, HR-SWI is ready to apply in current practice once a radiologist is trained. Until recently, there have been reports [14, 15] of high diagnostic performance of HR-SWI.

Thus, we hypothesized that although HR-SWI may have relatively poor diagnostic performance, but its accessibility is better than other imaging study including SMWI and N-3-fluoropropyl-2-carbomethoxy-3-4-iodophenyl nortropane (FP-CIT) Positron Emission Tomography (PET), so if there is good agreement between the reader’s diagnostic confidence and the probability which the final clinical conclusion and the HR-SWI imaging diagnosis match, it would be possible to reduce medical cost by reducing the number of patients requiring further imaging studies. So the purpose of this study was to evaluate the real-world diagnostic performance of the HR-SWI and investigate whether the reader’s predictions can be used to find cases which HR-SWI finding and final clinical diagnosis matched.

## Materials & methods

### Participants

This retrospective study was approved by the institutional review board of Soonchunhyang University Bucheon hospital and the requirement for written informed consent was waived. Since, the HR-SWI is non-invasive and does not require contrast media, we performed verbal informed consent after explaining that some additional time was added to MR examination in each patient. There were two groups in our study. One group included subjects who visited the outpatient clinic with suspected Parkinsonism, the other group included subjects who did not show Parkinsonism symptoms, but underwent brain MRI for other reasons. From April 2017 to January 2018, we collected data on all consecutive patients who underwent brain MRI including HR-SWI due to suspected Parkinsonism. All suspected Parkinsonism patients underwent FP-CIT PET and follow up care. The final clinical diagnosis was made during the follow up period based on the criteria for each disorder using the corresponding FP-CIT PET scan results, conventional MRI, and follow up clinical features observed by the neurologists [16, 17]. The Hoehn and Yahr (HY) scale and Unified Parkinson's Disease Rating Scale (UPDRS) were used to evaluate the severity of motor symptoms in Parkinson’s disease patients [18, 19]. The controls with non-Parkinsonism pathology group included the patients who visited the neurology or neurosurgery outpatient clinic between November 2017 and January 2018, older than 40 years, showed no signs or symptoms of Parkinsonism, and agreed to add HR-SWI to MR examination. And if there was neurodegenerative symptoms including cognitive impairment or sleep disorder [20, 21] during about 1–2 years follow-up period, the subject was excluded from the study considering the possibility of nigrostriatal degeneration and appropriate clinical managements were taken by neurologist or neurosurgeon.

### MRI protocol

MR imaging was performed on a 3T system (SIGNA HDx; GE Medical Systems, Milwaukee, WI, USA) with a 16-channel head coil. The imaging protocol included routine brain MRI and HR-SWI. We obtained the HR-SWI with axial plane parallel to the anterior commissure and posterior commissure. The detailed parameters were as follows: TR/TE: 52.2/25.24 ms, flip angle: 15, slice thickness: 1 mm, FOV: 20cm, matrix: 400 x 400 (in-plane resolution, 0.5 x 0.5mm), total acquisition time: 2:21.

### Imaging analysis

**Fig 1:**
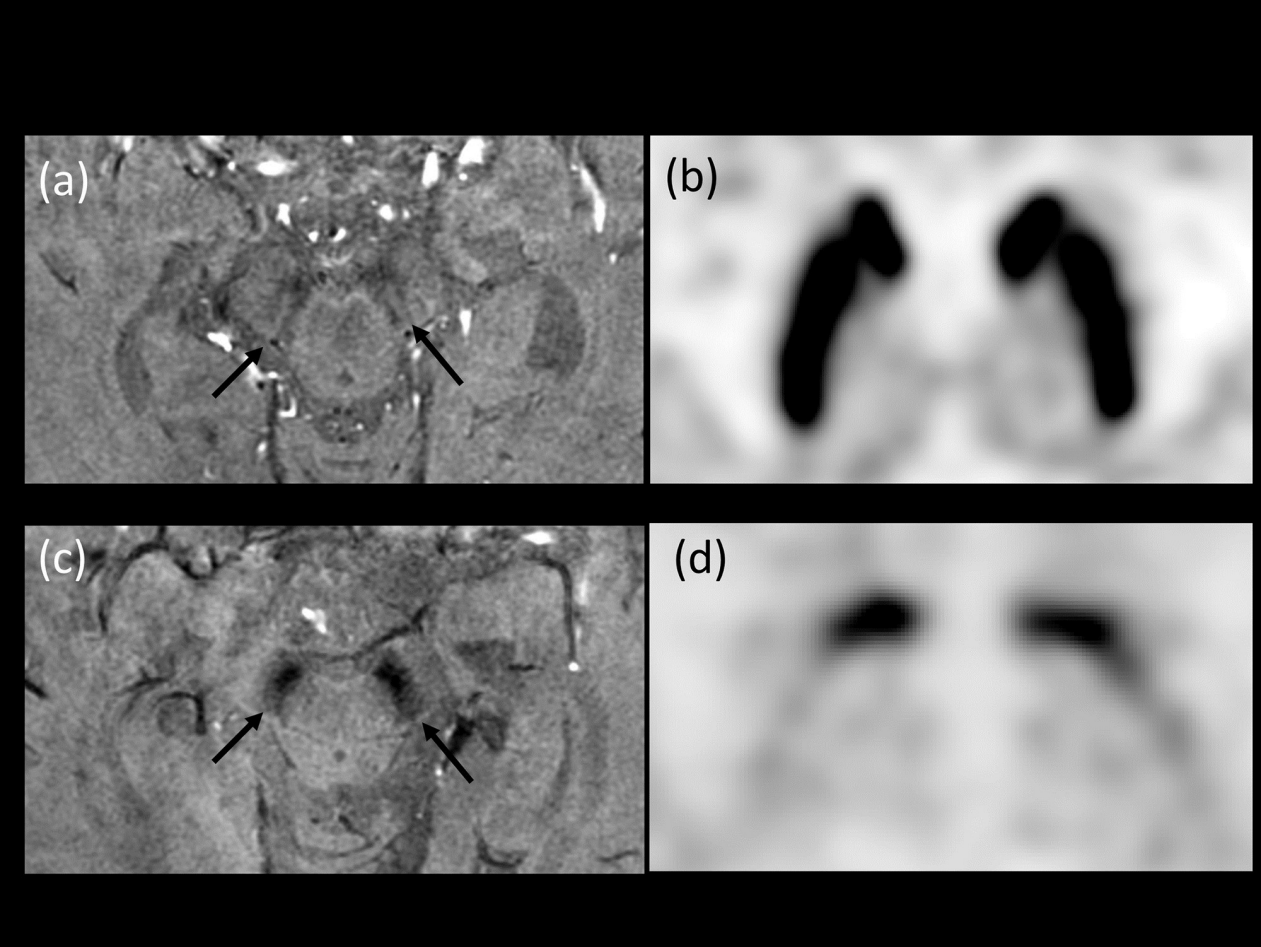
(a, b) An 82-year-old woman with gait disturbance. (a) High-resolution susceptibility-weighted imaging (HR-SWI) showed normal dorsolateral hypersignal intensity on both sides of the substantia nigra pars compacta (Black arrows, diagnostic confidence 99). (b) FP-CIT positron emission tomography (PET) showed normal binding on both sides of the putamen. The patient was assigned to the disease control group. (c, d) An 84-year-old woman with hand tremor. (c) HR-SWI showed poor visualization of dorsolateral hypersignal intensity in both sides of the substantia nigra pars compacta (Black arrows, diagnostic confidence 99). (d) FP-CIT PET showed decreased binding in both sides of putamen. The patient was diagnosed with idiopathic Parkinson’s disease (Hoehn and Yahr stage 5).

All MR imaging was retrospectively reviewed by two neuroradiologists (with two and eight years, respectively, of experience as neuroradiologists) without knowledge of FP-CIT PET findings or clinical features. The abnormalities of NG1 on HR-SWI were evaluated qualitatively by visual interpretation. On HR-SWI, the NG1 was defined as a structure with focal hypersignal intensity in the dorsolateral aspect of substantia nigra, which is surrounded by the hyposignal intensity[8, 9]. (Fig 1). Based on a previous study regarding normal NG1 on 7T MRI [3], NG1 was evaluated from the level of the inferior colliculi to the red nucleus of midbrain. Asymmetry of NG1 [22] and abnormal shape of the hypersignal intensity varying from the typical oval, linear to loop, cluster, and strip have been reported [12]. Therefore, regardless of the shape, the hypersignal intensity, which was noted in the dorsolateral aspect of substantia nigra, was described as normal NG1. Nigrostriatal degeneration was identified when there was no distinct hypersignal intensity within the dark signal intensity of the substantia nigra. After independent readings, the final diagnosis was made by agreement between the two readers. At that time, the two readers determined the diagnostic confidence score from 50 to 100% in a 0 to 100% scale (Not including the percentage range below to 0 to 50%, because the available diagnosis involves only two conclusions; normal or abnormal which in a purely random situation is 50%). When the diagnostic confidence score was 80 or lower, the major cause of decreased suspicion was described. The diagnostic confidence score was rated as follows: 50–60, limitation involved both NG1, hard to evaluate both NG1; 61–70, limitation affected one NG1, hard to distinguish true involvement or pseudolesion in one of NG1; 71–80, limitation mildly affected one of NG1, minor decrease in suspicion; 81–90, limitation involved one NG1, but easily able to distinguish limitation and does not affect final conclusion; 91–100, clearly visualized the entirety of both substantia nigra without any limitation (S1 Fig).

#### FP-CIT PET protocol and image analysis

FP-CIT PET images were obtained using a PET/CT scanner (Biograph mCT 128, Siemens Medical System) 180 min after the injection of 185 MBq 18F-FP-CIT. CT was performed at 80 kVp and 150 mAs with a slice thickness of 3.0 mm. The FP-CIT PET images were reconstructed with a TrueX algorithm and an all-pass filter using a 512 × 512 matrix. Visual analysis of FP-CIT PET images was performed by nuclear medicine specialists (J.P.H. and J.M.P., with 5 and 15 years of experience, respectively).

### Statistical analysis

The demographic data were compared using one-way ANOVA, chi-square, and Wilcoxon rank-sum tests. The interobserver agreement among the readers and imaging techniques (HR-SWI vs. FP-CIT PET) was calculated using Cohen’s kappa. The diagnostic performance of HR-SWI was evaluated by calculating its sensitivity, specificity, and accuracy. The receiver-operating characteristic (ROC) curve analysis was performed to determine the concordance between HR-SWI findings and final clinical diagnosis, according to the diagnostic confidence score. The optimal cut-off value was selected by Youden index. Statistical analysis was performed using MedCalc 18.2.1 (MedCalc Software, Belgium). P < 0.05 was considered statistically significant.

## Results

### Clinical characteristics of the participants

A total of 48 patients with suspected Parkinsonism underwent brain MRI including HR-SWI (13 men and 21 women, mean age: 68.5 ± 10.6 years, age range: 39–84 years, disease duration: 4.5 ± 5.4 years, range: l month– 22 years). The time interval between MRI with HR-SWI and FP-CIT PET was less than 3 months. All HR-SWI or FP-CIT PET images were used for the final analysis, including those of suboptimal image quality. The follow-up period from initial visit to finial clinical diagnosis was 2.7 ± 2.6 years (range 42 days– 11 years). Of the 48 patients who underwent testing, 31 were diagnosed with idiopathic Parkinson’s disease (iPD), and three with multiple systems atrophy (MSA; one with predominant cerebellar ataxia, and the remaining two with predominant Parkinsonism). These patients were classified as nigrostriatal degeneration group. Among the iPD patients, there were 17 patients with early stage of iPD with HY stage 0–2. The remaining 14 patients were included in the disease control group with various movement disorders. In the case of the controls with non-Parkinsonism pathology group, 9 patients who showed cognitive impairment and one patient who showed sleep disorder during follow-up at the outpatient clinic were excluded due to the possibility of nigrostriatal degeneration. A total of 21 subjects were included in the controls with non-Pakrinsonism pathology group (9 men and 12 women, mean age: 64.9 ± 9.1 years, age range: 45–80 years). The detailed clinical characteristics are described in Table 1. The HY stage distribution in iPD, final clinico-radiologic diagnosis of the disease control and the controls with non-Parkinsonism pathology were included in S1 Table.

**Table 1:** Demographics of the study population.

	Nigrostriatal degeneration^a^	Disease control	Non-PD control^b^	P value
	(n = 34)	(n = 14)	(n = 21)	
Age	68.5 ± 10.8	66.1 ± 13.9	64.9 ± 9.1	> 0.05^e^
M:F	13:21	3:11	9:12	> 0.05^f^
Disease Duration (years)	2 (1–6)	1 (0–7)	NA	> 0.05^g^
HY stage	1.5 (1–2.5)^c^	NA	NA	-
UPDRS	20.3 ± 11.8^d^			

Data are expressed as mean ± standard deviation for continuous variables and median (interquartile ranges) for non-normally distributed variables. Nigrostriatal degeneration^a^ included idiopathic Parkinson disease and MSA. Non-PD control^b^ refer the controls with non-Parkinsonism pathology group. ^c^Four and ^d^three patients were excluded from the analysis because the HY stage or UPDRS III was not available on electronic medical record. ^e^One way ANOVA for continuous variable with equal variances. ^f^Chi-square test for categorical variable. ^g^Wilcoxon rank-sum test for non-normal distribution.

M: male; F: female; HY: Hoehn and Yahr scale; UPDRS United Parkinson’s disease rating scale

#### Diagnostic performance of HR-SWI according to final clinical diagnosis and FP-CIT PET findings

Among the 69 patients included in our study, two were classified differently by the two readers. The interobserver agreement between the two readers was substantial (Kappa: 0.80, 95% CI: 0.66–0.94). After consensus reading, 25 patients were classified as true positive and 28 patients as true negative according to HR-SWI findings. The calculated diagnostic sensitivity, specificity, and accuracy of the consensus reading was 73.5%, 80.0%, and 76.8%, respectively (Table 2A). When analyzing only those who suspected Parkinsonism, the calculated specificity rose to 92.9% and 85.7% in the consensus reading, for reader 1 and reader 2, respectively. However, this change did not show a significant difference due to the overlap in 95% confidence interval between the overall results (Table 2B).

**Table 2:** Diagnostic performance of HR-SWI.

(A) All participants (n = 69)	Final clinical diagnosis		
	Nigrostriatal degeneration*	Control†	Total
Poor visualization of NG1 (Reader 1/Reader 2)	25 (25/25)	7 (6/9)	32
Good visualization of NG1 (Reader 1/Reader 2)	9 (9/9)	28 (29/26)	37
Total	34	35	69
Performance (%)	Sensitivity	Specificity	Accuracy
Reader 1 (95% confidence interval)	73.5 (55.6–87.1)	82.9 (66.4–93.4)	78.3 (66.7–87.3)
Reader 2 (95% confidence interval)	73.5 (55.6–87.1)	74.3 (56.4–87.5)	73.9 (61.9–83.8)
Consensus reading (95% confidence interval)	73.5 (55.6–87.1)	80.0 (63.1–91.6)	76.8 (65.1–86.1)
(B) Suspected Parkinsonism (n = 48)	Final clinical diagnosis		
	Nigrostriatal degeneration*	Disease control	Total
Poor visualization of NG1 (Reader 1/Reader 2)	25 (25/25)	1 (1/2)	26
Good visualization of NG1 (Reader 1/Reader 2)	9 (9/9)	13 (13/12)	22
Total	34	14	48
Performance (%)	Sensitivity	Specificity	Accuracy
Reader 1 (95% confidence interval)	73.5 (55.6–87.1)	92.9 (66.1–99.8)	79.2 (65.0–89.5)
Reader 2 (95% confidence interval)	73.5 (55.6–87.1)	85.7 (57.2–98.2)	77.1 (62.7–88.0)
Consensus reading (95% confidence interval)	73.5 (55.6–87.1)	92.9 (66.1–99.8)	79.2 (65.0–89.5)
(C) Concordance between HR-SWI and FP-CIT PET (n = 48)	FP-CIT PET finding		
	Decreased binding	Normal	Total
Poor visualization of NG1	26	0	26
Good visualization of NG1	10 (1/9)	12	22
Total	36	12	48

Nigrostriatal degeneration*† included idiopathic Parkinson disease and MSA. Control† included disease control and non-Parkinsonism control participants.

NG1: nigrosome 1; HR-SWI: high-resolution susceptibility-weighted imaging; PET: positron emission tomography

Of the 48 patients with suspected Parkinsonism, nine showed decreased binding of FP-CIT on the PET scan, but NG1 appeared normal in both sides on HR-SWI. Among them, only one patient was classified as disease control in final clinical diagnosis. One of the eight patients was diagnosed with MSA and seven had iPD. Six of seven iPD patients were early stage with HY stage 0–2. The interobserver agreement between HR-SWI and FP-CIT PET was moderate (Kappa: 0.57, 95% CI: 0.35–0.78).

### Analysis of diagnostic confidence score

**Fig 2:**
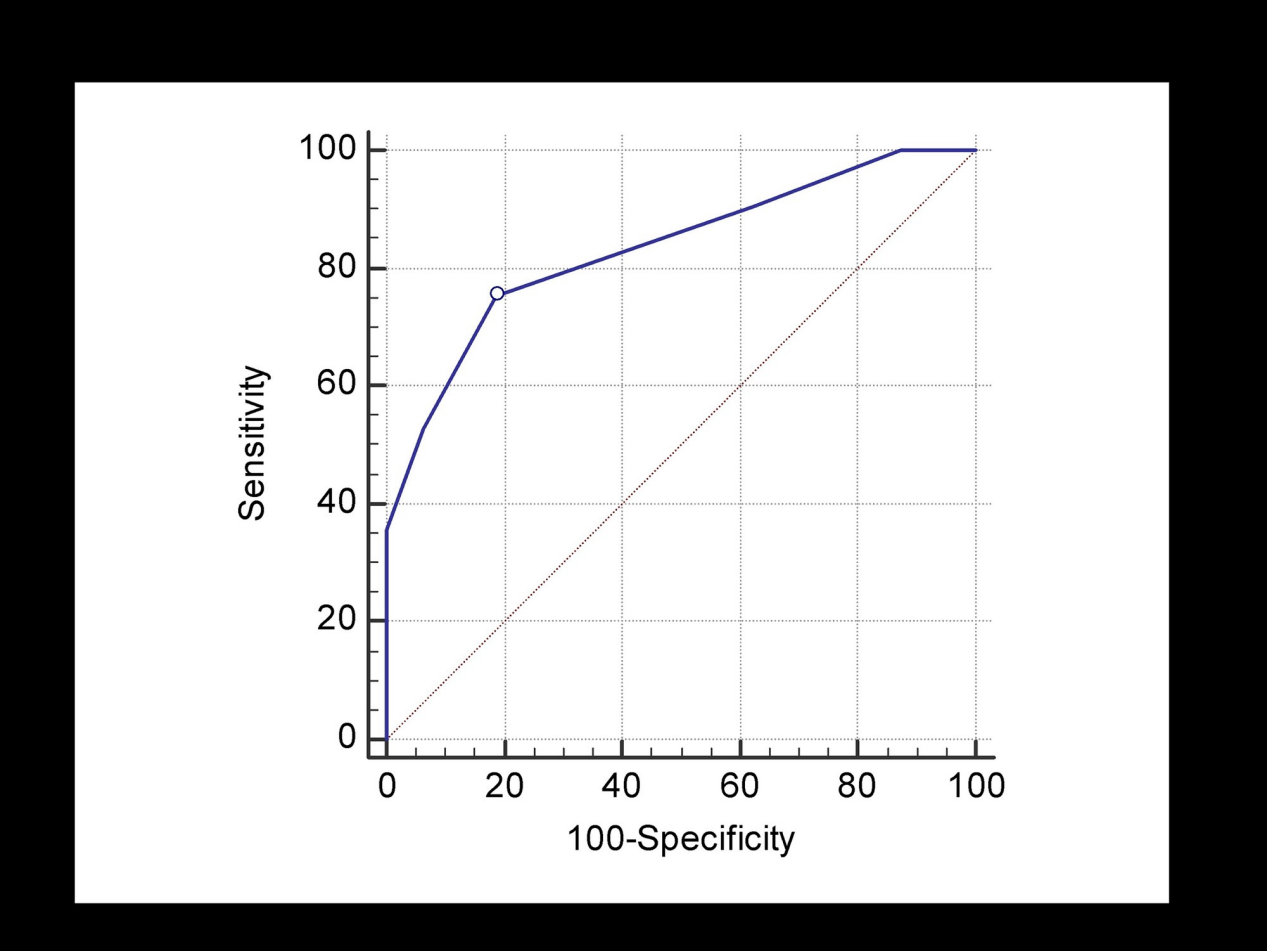
Receiving operating characteristic (ROC) curve of diagnostic confidence score to detect concordance case between high-resolution susceptibility-weighted imaging (HR-SWI) and final clinical diagnosis. The circle represents the cut-off point calculated by Youden index (Score > 70, Sensitivity: 73.5%, Specificity: 80.0%, Accuracy: 76.8%).

**Fig 3:**
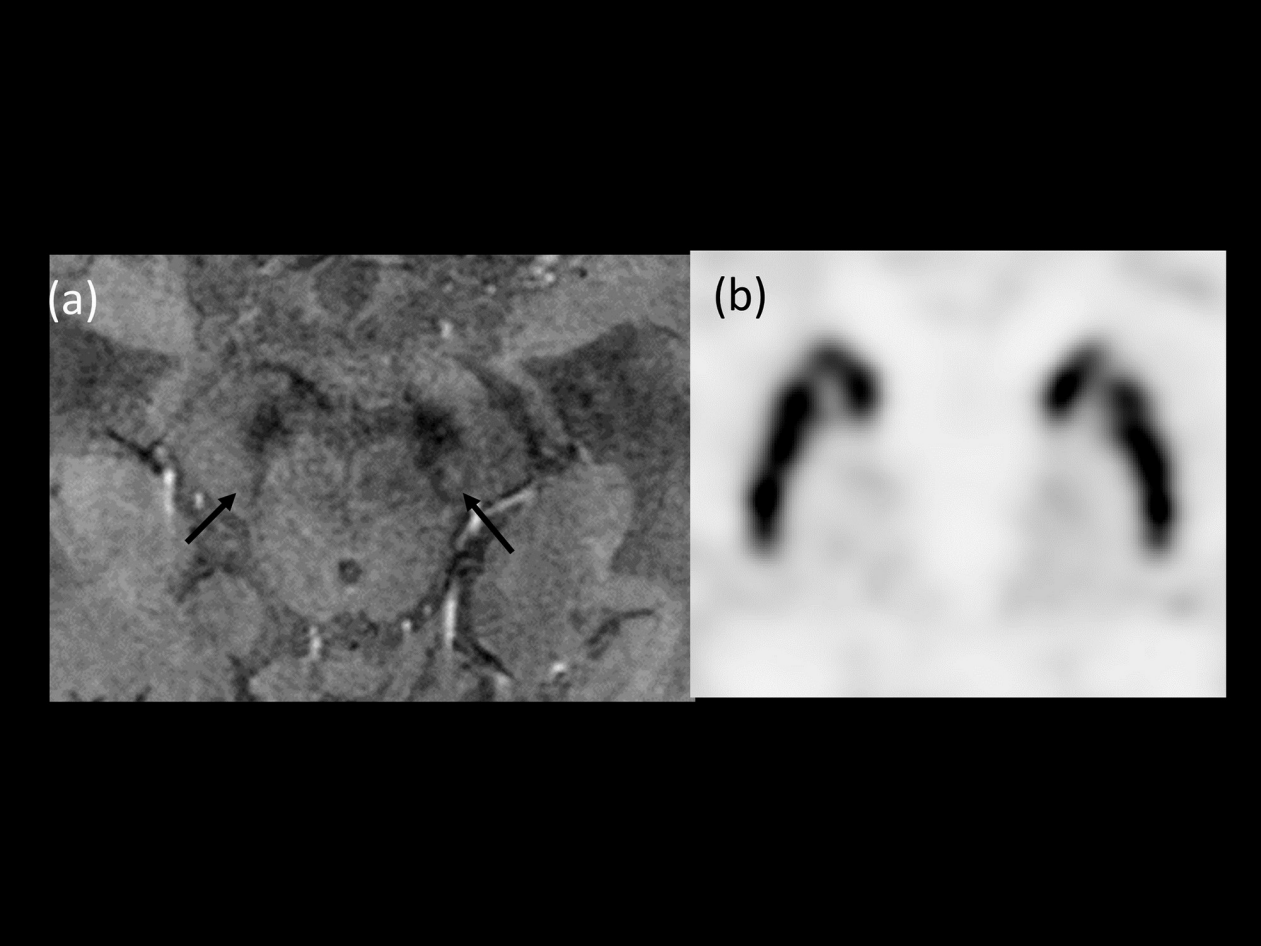
An 82-year-old man with gait disturbance. (a) HR-SWI showed asymmetry in both substantia nigra pars compacta (Black arrows, diagnostic confidence 90; The suspicion was not affected by asymmetry of right side due to hypersignal intensity area in the right dorsolateral aspect of substantia nigra, regardless of shape). (b) FP-CIT PET revealed normal binding in both sides of the putamen. The patient was assigned to the disease control group and the final clinical diagnosis was normal pressure hydrocephalus (NPH).

**Fig 4:**
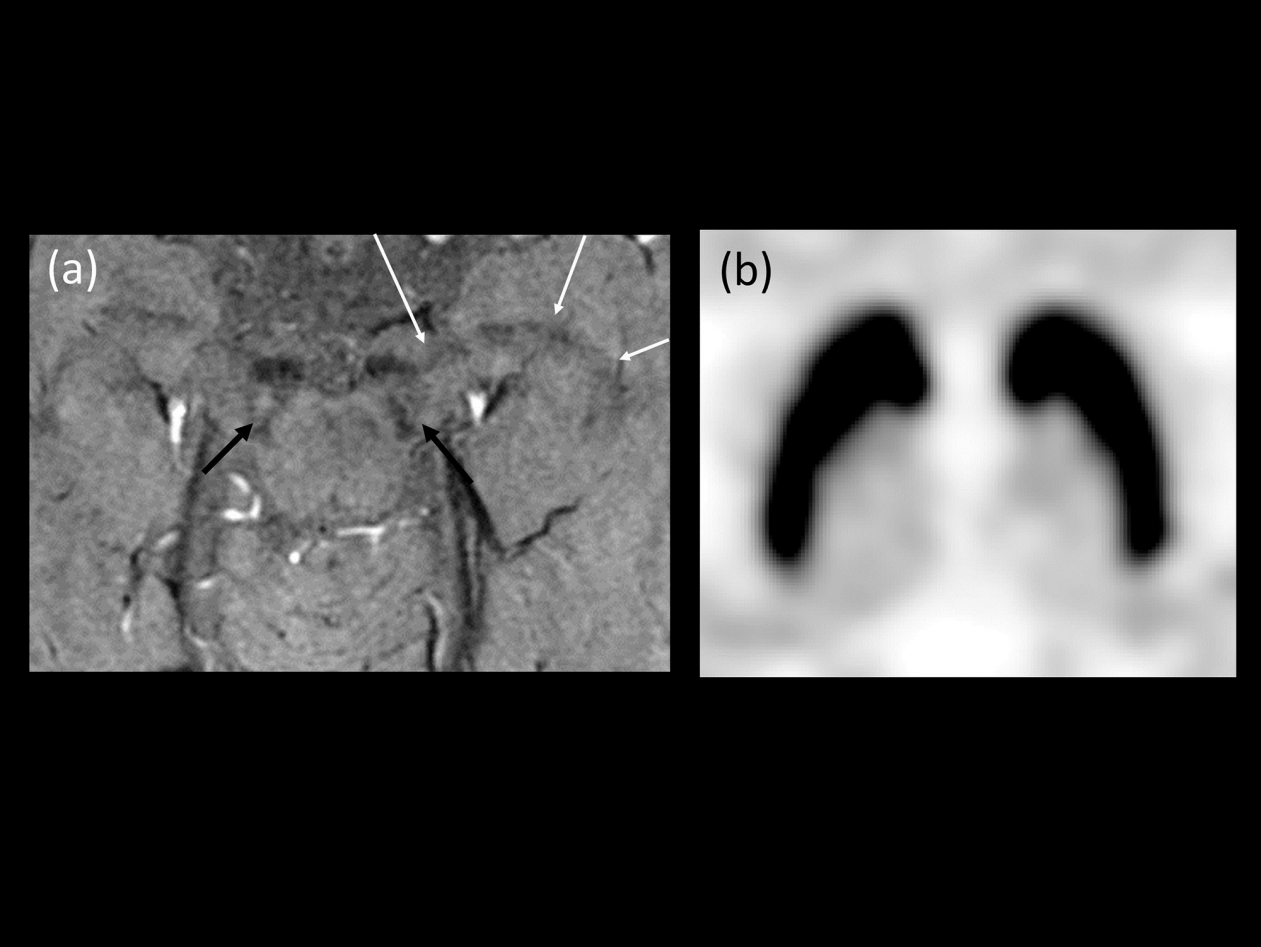
An 80-year-old woman with right hand and severe head tremor. (a) HR-SWI showed a curvilinear dark signal intensity (indicated with white arrows; diagnostic confidence 80; although the motion artifact included the left NG1 area, the normal dorsolateral hypersignal intensity was relatively well preserved with a minor decrease in suspicion.) involving the left medial temporal lobe and left side of the substantia nigra. The normal dorsolateral hypersignal intensity was relatively preserved except the artifact-affected areas (Black arrows). (b) Both sides of the putamen showed normal binding on FP-CIT PET and the patient was diagnosed with essential tremor.

**Fig 5:**
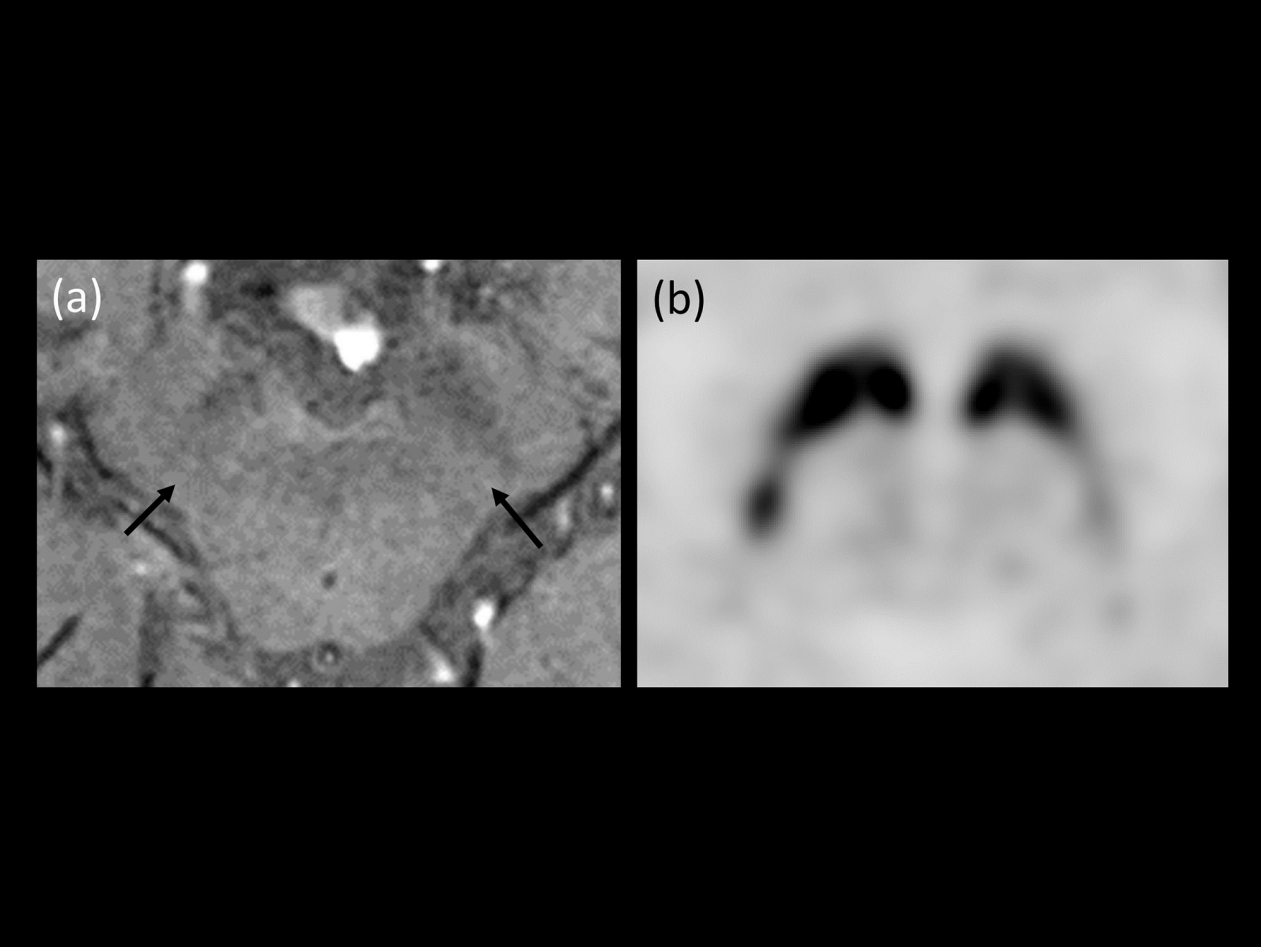
A 55-year-old woman with gait disturbance and right arm tremor. (a) HR-SWI showed poor mineralization of bilateral substantia nigra pars compacta (Black arrows, diagnostic confidence 60; Poor mineralization of the substantia nigra limited the evaluation of both NG1 areas). (b) FP-CIT PET showed decreased binding on the left side of the putamen. The patient was diagnosed with idiopathic Parkinson’s disease (HY stage 1.5).

**Fig 6:**
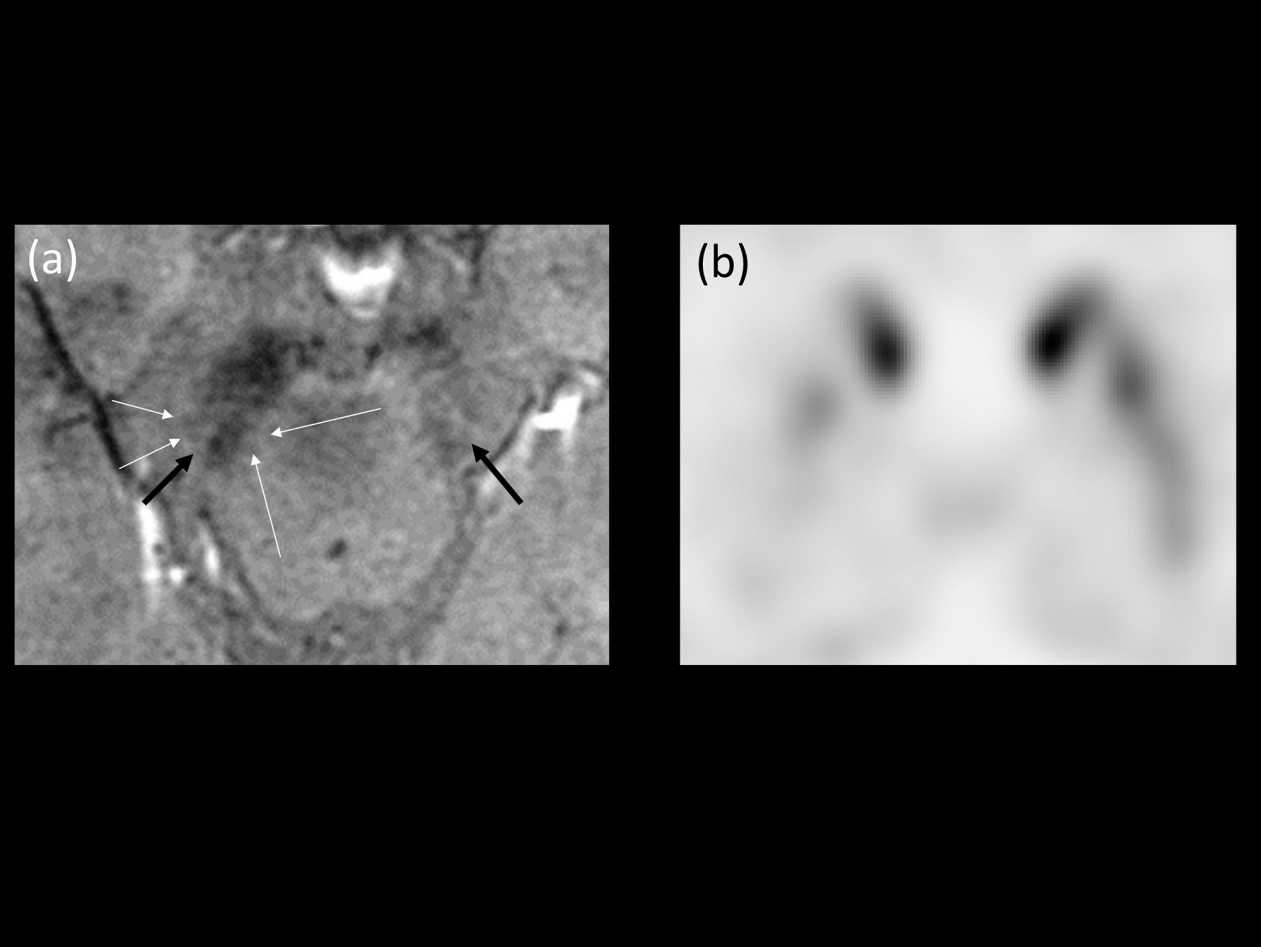
A 69-year-old woman with left hand tremor. (a) HR-SWI showed a curvilinear dark SI structures in the right substantia nigra (White arrows). We presumed the normal dorsolateral hypersignal intensity was affected by partial volume averaging by the venous structures (Black arrows, diagnostic confidence 70; partial volume averaging artifact involved left NG1 area, resulting in decreased suspicion). (b) However, the FP-CIT PET showed decreased binding on the both side of putamen, more prominent in the right side. The patient was diagnosed with idiopathic Parkinson’s disease (HY stage 2).

The mean value of diagnostic confidence for 69 patients was 81.1 ± 14.6. The calculated AUC on the ROC curve of diagnostic confidence score was 0.83 (95% CI: 0.72–0.91, p < 0.05, Fig 2). The cutoff value for indication of concordant cases was >70. At this value, the sensitivity was 75.5% (95% CI: 61.7–86.2) and specificity was 81.3% (95% CI: 54.4–96.0). Of 69 patients, 43 scored over 70 for diagnostic confidence and among them, only 3 patients were discordant between consensus reading and final clinical diagnosis. The causes of reduced scores were asymmetry, motion artifacts, poor mineralization, and partial volume averaging artifacts (Table 3, Figs 3–6). Asymmetry was detected in 10 patients, four of which were diagnosed with early idiopathic Parkinson’s disease, HY stage 1–2. In the case of scan images with motion artifacts, five of the six subjects were disease controls or the controls with non-Parkinsonism pathology; and among the subjects, three subjects presented tremors including head tremors. The poor mineralization and partial volume averaging artifact groups contained participants from both the nigrostriatal degeneration and control groups.

**Table 3:** Causes of reduced diagnostic confidence (diagnostic confidence < 80).

Cause	n (Nigrostriatal degeneration/control)
Asymmetry	10 (7/3)
Motion artifact	6 (1/5)
Poor mineralization	6 (4/2)
Partial volume	4 (2/2)
Total	26 (14/12)

## Discussion

This study showed that 3T HR-SWI had an acceptable range of sensitivity, specificity, and accuracy of NG1 detection for the diagnosis of nigrostriatal degeneration, including idiopathic Parkinson disease and Parkinson-plus syndromes. The HR-SWI finding showed substantial agreement with FP-CIT PET. Of the nine mismatch cases, HR-SWI detected one FP-CIT PET false positive case, but there were eight HR-SWI false negative cases, 6 of which were early stage of the iPD. By using the diagnostic confidence score, we were able to detect cases that could be easily diagnosed on HR-SWI. We also demonstrated the possible causes that reduce the diagnostic confidence. Our results indicate that although it may be challenging to replace FP-CIT PET with HR-SWI, but add value to the diagnosis MR protocol of suspected Parkinsonism.

Previous studies using HR-SWI in the diagnosis of Parkinson’s disease have highlighted its diagnostic performance, but there are few studies that evaluated its clinical added value [6–9, 15]. Moreover, its diagnostic performance could be overestimated by excluding suboptimal images in exploratory settings. Our results showed relatively low diagnostic performance compared with similar previous studies using HR-SWI in the diagnosis of Parkinson’s disease [6–8, 15]. One possibility for this discrepancy is that we did not exclude patients from the study with poor mineralization in the substantia nigra, images with mild motion artifacts, or partial volume averaging artifacts. Instead of exclusion, suboptimal images received low diagnostic confidence scores. In previous studies, the percentage of images excluded from evaluation due to motion artifacts has been reported to be approximately 5.3–13.2%, which is similar to the number of motion artifact images in our study (8.7%). However, in patients with clinically suspected Parkinsonism including those with essential tremor, which need to be subsequently excluded from the Parkinsonism group, motion artifacts are hard to avoid due to tremors in the head and jaw [23]. Approximately 10% of images could be affected by tremors, which should be recognized when considering the real world application of HR-SWI in the diagnosis of Parkinson’s disease. Patients with poor mineralization in the substantia nigra were also not excluded from our study. The degree of mineralization of the substantia nigra differs from person to person and is not affected by age [22, 24]. As decreased mineralization in the substantia nigra limits NG1 evaluation by HR-SWI, previous studies excluded these patients from analysis [15]. In our study, poor mineralization of NG1 was detected in both disease control and suspected Parkinsonism patients. Therefore, this result may indicate that patients with decreased mineralization in substantia nigra should be referred to other modalities such as FP-CIT PET or neuromelanin imaging [25, 26] because there is a limitation in evaluation by HR-SWI. The other possible reason for discrepancy of diagnostic performance is that the patients included in our study had relatively short disease durations or were in a lower HY stage (duration: 4.7 ± 5.3 years; HY stage: 1.5, 1–2.5) compared to previous studies [6, 7, 15].

Asymmetry in substantia nigra also contributed to lower confidence scores in our study. In a previous study using 7T MRI for healthy volunteers, 24% showed different visibility [22]. However, there are studies that show that asymmetry in early stage Parkinson disease (HY stage 1–2) is correlated with laterality [11, 15]. The group of participants with low diagnostic confidence scores due to substantia nigra asymmetry consisted of participants with early stage Parkinson’s disease with asymmetry and false positive cases. In the current study, we analyzed the images blind to clinical conditions. In clinical practice, knowledge of the patient’s HY stage and the laterality of the motor symptoms may be helpful in HR-SWI analysis.

In subgroup analysis with suspected Parkinsonism group only, the higher specificity was noted compared to the results from all subjects. This difference was resulted from lower diagnostic performance in control with non-Parkinsonism pathology patients (True negative: False positive; 15:6) than disease control (True negative: False positive; 13:1). It was too small number of false positive cases to detect any significant trend based on this result. But when performing HR-SWI in subject without Parkinsonism feature, the readers should aware of possibility of false positive.

**Fig 7:**
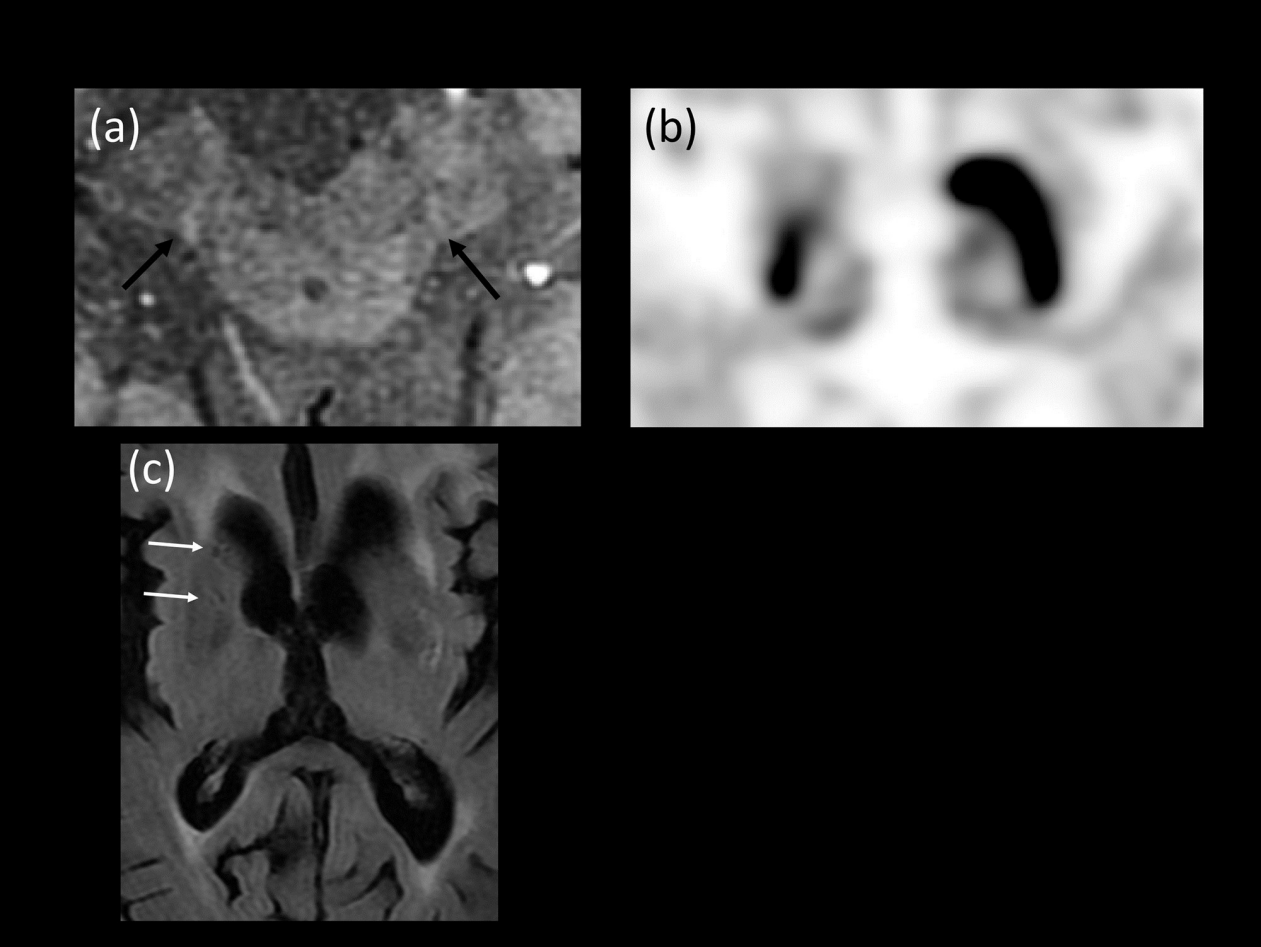
A 76-year-old woman with right hand tremor. (a) High-resolution susceptibility-weighted imaging (HR-SWI) revealed normal dorsolateral hypersignal intensity in bilateral substantia nigra pars compacta (Black arrows, diagnostic confidence 60; poor mineralization involved both substantia nigra, which limited the evaluation of bilateral NG1). (b) FP-CIT positron emission tomography (PET) showed decreased binding in the right side of the putamen. (c) Fluid attenuated inversion recovery (FLAIR) imaging revealed miniscule, old lacunar infarctions in the right caudate head and putamen (indicated by white arrows). The patient was assigned to the disease control group and the final clinical diagnosis was vascular Parkinsonism.

The diagnostic agreement between FP-CIT PET and HR-SWI was moderate. There were 9 SWI-PET discordant cases, of which only one case matched between HR-SWI finding and final clinical diagnosis (Fig 7). Interestingly, in this case, FP-CIT PET showed decreased binding in the right caudate nucleus and putamen. However, in the HR-SWI, NG1 was visualized relatively well and small, old infarctions of the caudate nucleus and putamen were observed on conventional MRI. We therefore suspected vascular Parkinsonism in this patient, which is known to be difficult to diagnose by imaging studies [27]. Thus, confirmation of the absence of nigrostriatal degeneration using HR-SWI may be helpful in the diagnosis of vascular Parkinsonism [28]. All others eight mismatch cases were HR-SWI false negative cases and 6 cases were early stage iPD patients with unilateral NG1 involvement. This finding may be suggesting that HR-SWI may have relatively lower sensitivity in early stage iPD than FP-CIT PET. One hypothesis of this lower sensitivity is that asymmetry can be seen in normal subjects, as described above. The other is that HR-SWI, which evaluate NG1, should visualize relatively smaller structures compared to FP-CIT PET, which evaluate putamen. Therefore, the reader should be careful when asymmetry is noted on HR-SWI in the patient who suspected early stage of iPD.

Using the diagnostic confidence score, we were able to identify the patients with a high likelihood of case who the HR-SWI finding- the final clinical diagnosis match. The confidence score cut-off was >70 in this study, and 43 patients were eligible. Only three of them had discrepancies between SWI finding and final clinical diagnosis (retrospectively, 2 case showed asymmetry and 1 showed partial volume averaging). This indicates that the prediction of the reader is relatively well correlated with the clinical diagnosis. The diagnostic confidence score of this study can be used in daily practice by replacing words such as probably or possible (for example, confidence score 80–90, and 50–60, respectively), which indicate the degree of suspicion. MRI should be performed to exclude structural lesions such as subdural hemorrhages or normal pressure hydrocephalus (NPH) in patients with suspected Parkinsonism, and adding HR-SWI to MRI protocol can be helpful to reduce medical cost and radiation dose for FP-CIT PET [1]. Moreover, FP-CIT PET requires cyclotrons to make nuclear isotope, so it is less accessible than 3T MRI in difficult traffic areas. For instance, in the case of advanced iPD, these patients have poor functional status, going out, including a hospital visit for examination can be painful and wasting. According to our results, these patients have a relatively low frequency of NG1 asymmetry which causes false negative on HR-SWI. The HR-SWI may be able to increase the convenience of these patients if the diagnose can be made by MRI including HR-SWI alone without preparation of nuclear isotope.

This study has several limitations. First, although we used strict criteria for clinical diagnosis, there is a possibility of misdiagnosis. However, we had a follow up period in order to strengthen the diagnosis in the suspected Parkinsonism group and healthy volunteers. In addition, although FP-CIT PET was not performed in the healthy volunteer group, considering the prevalence of Parkinson disease in North America, which is approximately 572/100000 [29], the possibility of including asymptomatic Parkinson disease patients in the control group is not expected to be significant. Second, we did not use tailored protocols such as SMWI [30] as it currently has limitations in the application of routine practices due to post processing. However, since the present study has proven to have added value with HR-SWI in the evaluation of Parkinson’s disease, the cost-benefit of using the SMWI, which is known to have higher diagnostic performance, could be evaluated in further studies. Third, the number of subjects was relatively small and the study was performed with retrospective design. Therefore, the results of this study need to be confirmed in a prospective study with larger cohort.

## Conclusion

The diagnostic performance of NG1 detection on HR-SWI with 3T MRI was within the acceptable range for the detection of nigrostriatal degeneration. We summarized several causes of reducing the diagnostic confidence of HR-SWI, and the reader's diagnostic confidence helped to find cases which HR-SWI finding and final clinical conclusion match. So HR-SWI may be of added value in the evaluation of suspected Parkinsonism.

## Supporting information

S1 Table Clinical diagnosis or cause of MRI in study subjects. (DOCX)

S1 Fig Diagnostic confidence score 50. (DOCX)

S2 Fig Diagnostic confidence 70. (DOCX)

S3 Fig Diagnostic confidence 80. (DOCX)

## Abbreviations

ANOVA: Analysis of Variance
AUC: Area Under the Curve
CT: Computed Tomography
FP-CIT: N-3-fluoropropyl-2-carbomethoxy-3-4-iodophenyl nortropane
HR-SWI: High resolution susceptibility weighted image
HY stage: Hoehn and Yahr stage
iPD: idiopathic Parkinson disease
MRI: Magnetic Resonance Imaging
MSA: Multiple System Atrophy
NG: Nigrosome
PD: Parkinson disease
PET: Positron Emission Tomography
ROC curve: Receiver Operating Characteristic curve
UPDRS: Unified Parkinson’s disease Rating Scale
