# Supplementary material for: The clinical application of nigrosome 1 detection on high-resolution susceptibility-weighted imaging in the evaluation of suspected Parkinsonism: The real-world performance and pitfalls
Source: PLoS One. 2020 Apr 2;15(4):e0231010. doi: 10.1371/journal.pone.0231010 (PMC7117705; doi:10.1371/journal.pone.0231010)
Supplement: S1 Table — (DOCX) [file pone.0231010.s001.docx]

**S1 Table. Clinical diagnosis or cause of MRI in study subjects.**

| Initial 79 subjects and final 69 subjects after exclusion of 10 subjects . | | | | |  |
| --- | --- | --- | --- | --- | --- |
| Nigrostriatal degeneration (n=34) | | |  | Disease control (n=14) | |
| Final clinical diagnosis | N | Final clinical diagnosis | | | N |
| Idiopathic Parkinson disease | 31 | Vascular dementia | | | 1 |
| - HY 0 | 3 | Normal pressure hydrocephalus | | | 2 |
| - HY 1 | 9 | Myoclonus | | | 1 |
| - HY 1.5 | 3 | Autoimmune encephalitis (anti-Yo Ab) | | | 1 |
| - HY 2 | 2 | Vascular Parkinsonism | | | 1 |
| - HY 2.5 | 7 | Delirium | | | 1 |
| - HY 3 | 2 | Drug induced Parkinsonism | | | 3 |
| - HY 4 | 3 | Essential tremor | | | 2 |
| - HY 5 | 2 | Jaw dystonia | | | 1 |
| - NA | 3 | Psychogenic tremor | | | 1 |
| MSA | 3 |  | | |  |
| Healthy volunteer (n=21) | | | | Exclusion (n=10) | |
| Cause of MRI | | | N | Clinical diagnosis | N |
| Transient Ischemic Attack | | | 4 | Vascular dementia | 4 |
| Headache | | | 4 | Alzheimer disease | 4 |
| Dizziness | | | 3 | Mild cognitive impairment | 1 |
| Temporal lobe epilepsy | | | 1 | Sleep disorder | 1 |
| Guilain-Barre syndrome | | | 1 |  |  |
| F/U for subdural hematoma | | | 2 |  |  |
| Lacunar infarction | | | 1 |  |  |
| Vestibular schwannoma | | | 1 |  |  |
| Benign paroxysmal positional vertigo | | | 1 |  |  |
| Vertebrobasilar insufficiency | | | 1 |  |  |
| Carotid artery stenosis | | | 1 |  |  |
| Brain metastasis work up(supratentorial dot-like) | | | 1 |  |  |
