# Supplementary material for: The clinical application of nigrosome 1 detection on high-resolution susceptibility-weighted imaging in the evaluation of suspected Parkinsonism: The real-world performance and pitfalls
Source: PLoS One. 2020 Apr 2;15(4):e0231010. doi: 10.1371/journal.pone.0231010 (PMC7117705; doi:10.1371/journal.pone.0231010)
Supplement: S1 Fig — (DOCX) [file pone.0231010.s002.docx]

**S1 Fig. Diagnostic confidence score 50.**

**
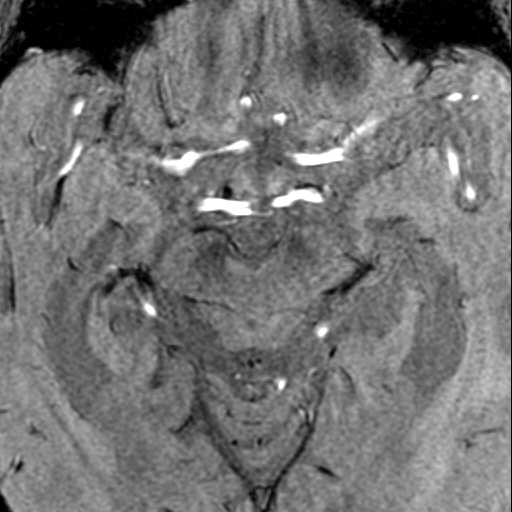
**

A 77-year-old woman with arm and head tremor. On HR-SWI, there were ill-defined linear low SI along the phase encoding direction (white arrows) and increased background noise through entire image. We concluded motion artifact affected bilateral NG1 (black arrows) and hard to evaluate NG1 by this image. The patient was diagnosed as autoimmune encephalitis with anti-Yo antibody positive.
