# Supplementary material for: The clinical application of nigrosome 1 detection on high-resolution susceptibility-weighted imaging in the evaluation of suspected Parkinsonism: The real-world performance and pitfalls
Source: PLoS One. 2020 Apr 2;15(4):e0231010. doi: 10.1371/journal.pone.0231010 (PMC7117705; doi:10.1371/journal.pone.0231010)
Supplement: S2 Fig — (DOCX) [file pone.0231010.s003.docx]

**S2 Fig. Diagnostic confidence 70.**

**
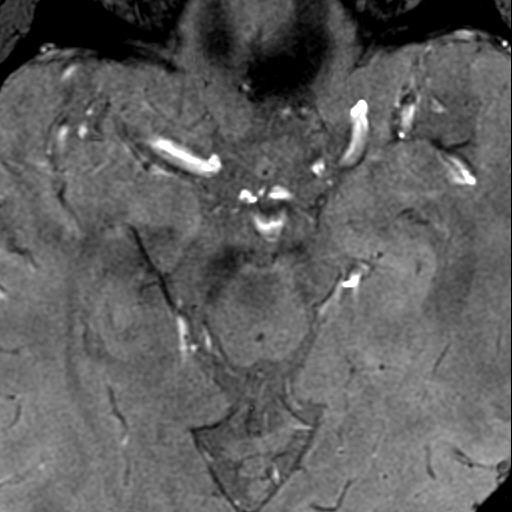
**

A 69-year-old woman with hand tremor. On HR-SWI, there were sharp thin linear dark SI structure were overlapped (white arrows) with right NG1. Left NG1 was relatively well visualized (black arrow). We concluded partial volume artifact of vascular structure affected right NG1 and it was hard to conclude true nigrostriatal degeneration or pseudolesion. The patient was diagnosed as idiopathic Parkinson disease (HY stage 1, left tremor).
