# Supplementary material for: The clinical application of nigrosome 1 detection on high-resolution susceptibility-weighted imaging in the evaluation of suspected Parkinsonism: The real-world performance and pitfalls
Source: PLoS One. 2020 Apr 2;15(4):e0231010. doi: 10.1371/journal.pone.0231010 (PMC7117705; doi:10.1371/journal.pone.0231010)
Supplement: S3 Fig — (DOCX) [file pone.0231010.s004.docx]

**S3 Fig. Diagnostic confidence 80.**


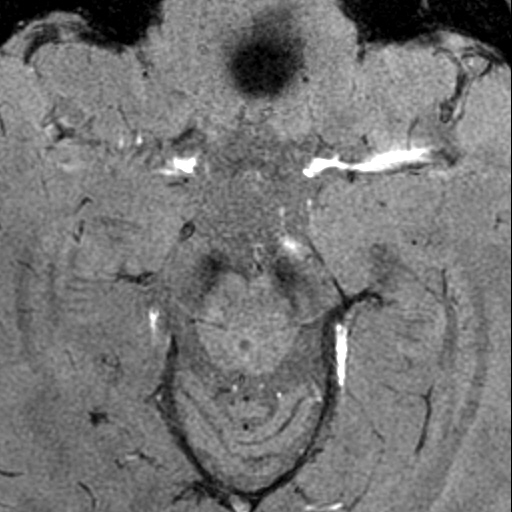


A 48-year-old man with gait disturbance. On HR-SWI, Asymmetrical SWI low signal intensity was noted in the right NG1 area. As there was no definite normal hypersignal intensity in the right NG1 area, we presumed that there was nigrostriatal degeneration involvement of the right side. However, comparing with dark signal intensity of substantia nigra, there was intermediate signal intensity area (white arrow) at the opposite side of the relatively normal-looking left NG1 area (black arrow). So our suspicion of conclusion was decreased, but not significantly affected by that finding. On FP-CIT PET, there were decreased bindings in the bilateral putamen and the patient was diagnosed as MSA-C type.
